# What Are the Personality Types Among Emergency Medicine Physicians?

**DOI:** 10.7759/cureus.14959

**Published:** 2021-05-11

**Authors:** Miles M Hunter, Catherine Patocka

**Affiliations:** 1 Department of Emergency Medicine, Cumming School of Medicine, University of Calgary, Calgary, CAN

**Keywords:** leadership, interpersonal and communication skills, conflict management, personality type, insights discovery

## Abstract

Introduction

Emergency medicine physicians work in high-stress environments that strain interpersonal skills, communication, and decision-making. Personality profile assessment tools have been used in educating the corporate world to enhance self-awareness, improve communication, and decrease conflict. Despite this, personality profile assessment tools have not been applied extensively within the emergency department context. As such, we explored whether Insights Discovery (Insights, Dundee, Scotland), a registered personality assessment tool, could contribute valuable understanding into the personality landscape of emergency medicine physicians and help tailor future educational interventions.

Methods

A cross-sectional survey was conducted via online administration of the Insights Discovery questionnaire to 30 attending emergency physicians of urban tertiary-care and community emergency departments of Calgary, Alberta, Canada.

Results

A disproportionately low number of fiery red personality types, typically described as competitive and strong-willed, existed among the study groups. No other significant differences were found between the proportions of other personality types or between physician characteristics such as gender or years of experience.

Conclusion

This study sheds early light on the personality characteristics of physicians within the emergency department environment, which may help individuals and departments tailor interventions to improve interpersonal communication and interactions.

## Introduction

Medical specialty stereotypes are often created [1], but their validity is not well-established [2]. Personality assessment tools are a more objective way to ascertain an individual's method of expressing and relating to their environment and peers [3]. These tools have been used to improve self-assessment, communication, and team dynamics in the corporate world, but they are rarely employed in medicine [4,5]. Furthermore, emergency medicine physicians work in high-stress environments that strain interpersonal skills, communication, and decision-making. This gap in physician self-assessment is at odds with the desire among physicians and emergency departments to improve key competencies, including collaboration, leadership, and communication skills [6]. A greater understanding of physician personality traits may lead to interventions that help foster and harness these crucial competencies within our fast-paced and unique environment.

Existing literature in this area is limited. Only a small Australian cohort of senior leadership emergency medicine staff [4] and a group of German emergency medicine conference attendees have been studied [5]. Personality profiles in these studies were assessed using the Myers-Briggs Test Indicator and Hamburg Personality Inventory, respectively. These instruments stratify people into up to 16 distinct personality profiles which can make interpretation cumbersome and non-intuitive.

In contrast, Insights Discovery (Insights, Dundee, Scotland) is a registered and validated tool that arranges four personality profiles under the colors of cool blue, fiery red, sunshine yellow, and earth green [7]. These color headings provide clear and memorable communication of results to users. To date, emergency department physicians in North America have not been assessed using a simple, easy-to-communicate personality tool such as Insights Discovery. In our study, we applied the Insights Discovery tool to attending (also known as "staff") emergency physicians to assess the feasibility of this personality tool applied in the emergency department setting. We hypothesized that a broad landscape of each personality color would be represented among the emergency physician cohort.

## Materials and methods

This study was approved by the Conjoint Health Research Ethics Board of the University of Calgary in Calgary, Alberta, Canada (Ethics ID: REB19-0644) and carried out with permission from Insights Discovery at the University of Calgary.

Study design, setting, and population

A cross-sectional survey design was employed between June and September of 2019 in the urban tertiary-care and community emergency departments of Calgary, Alberta, Canada. Staff emergency medicine physicians were contacted via email (obtained with permission) and provided with study information, informed consent, and instructions for self-enrollment.

Intervention

**Figure 1:**
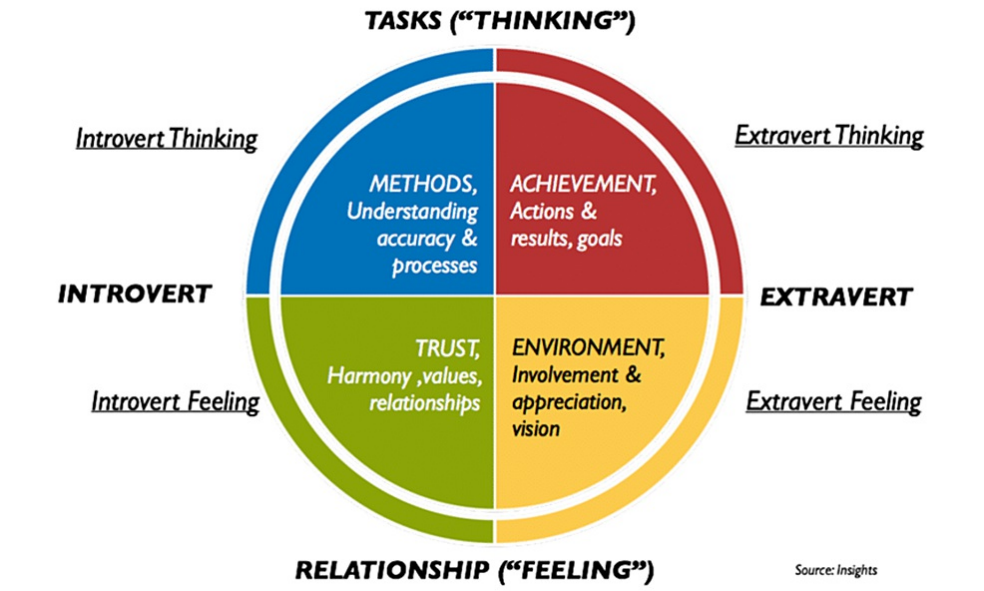
Pictorial representation of the Insights Discovery personality colors.

The Insights Discovery questionnaire is a psychometric assessment tool that uses 25 statements surrounding preferences in behavioral patterns where each statement provided four multiple-choice answers to form a total of 100 possible word pairs. The word pairs chosen by participants are distilled down to a label of one of four colors to represent the participants' observable behavioral patterns. The four colors include fiery red (extravert thinking), cool blue (introvert thinking), sunshine yellow (extravert feeling), and earth green (introvert feeling). Insights Discovery is a registered test of The British Psychological Society that has demonstrated construct validity, internal reliability, and temporal consistency [7]. Further descriptions of the characteristics of each color are represented pictorially within Figure 1 [8].

The Insights Discovery questionnaire was emailed to participants for online completion, with consent implied by voluntary participation. Questionnaire completion required an estimated 15-25 minutes. Once completed, participants attended an in-person debrief with Insights Discovery, where they received their personality profile assessment and a two-hour workshop on understanding their personality profile.

Outcome measures

The primary outcome was the proportion of each primary personality color among emergency physicians. Secondary outcomes included subgroup distribution of colors for male compared to female physicians and clinical experience of less than compared to greater than 10 years.

Data analysis

Primary outcome results are reported as proportions with respective 95% confidence intervals. The null hypothesis for this study was equal to 25% proportions of each of the four respective personality colors. Secondary outcome results were compared using the chi-squared test for categorical data. A p-value of 0.05 was set to determine statistically significant differences.

Sample size

A convenience sample of 35 physicians was contacted, with 30 physicians enrolled to achieve proof-of-concept and feasibility of the study.

## Results

**Figure 2:**
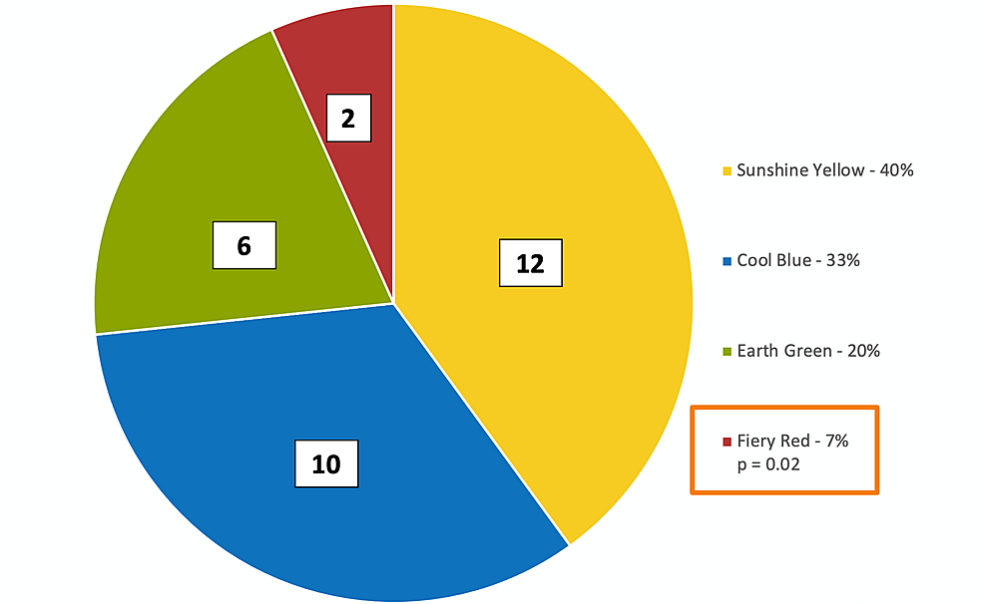
Proportion of physicians with each personality color according to the Insights Discovery questionnaire.

All 30 of the enrolled physician participants completed the study, with a review completed by the authors to ensure no duplicate responses were recorded. The proportion of physicians with each personality color is represented in Figure 2. Twelve of 30 participants (40%) were sunshine yellow, 10 (33%) were cool blue, six (20%) were earth green, and two (7%) were fiery red. Disproportionate results (null hypothesis of equal to 25% proportions for each color) were only statistically significant for fiery red (p=0.02, 95% CI 0.9-22.5%).

**Figure 3:**
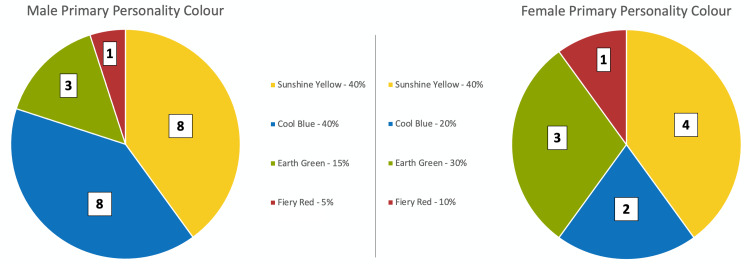
Proportion of male and female physicians with each personality color according to the Insights Discovery questionnaire.

Secondary outcomes compared male and female participants, as well as those with less than and greater than 10 years of clinical experience. Among male physician participants, eight of 20 participants (40%) were sunshine yellow, eight (40%) were cool blue, three (15%) were earth green, and one (5%) was fiery red. Among female participants, four of 10 participants (40%) were sunshine yellow, two (20%) were cool blue, three (30%) were earth green, and one (10%) was fiery red. As shown in Figure 3, male compared to female physicians showed no statistical significance between groups for each respective personality color.

**Figure 4:**
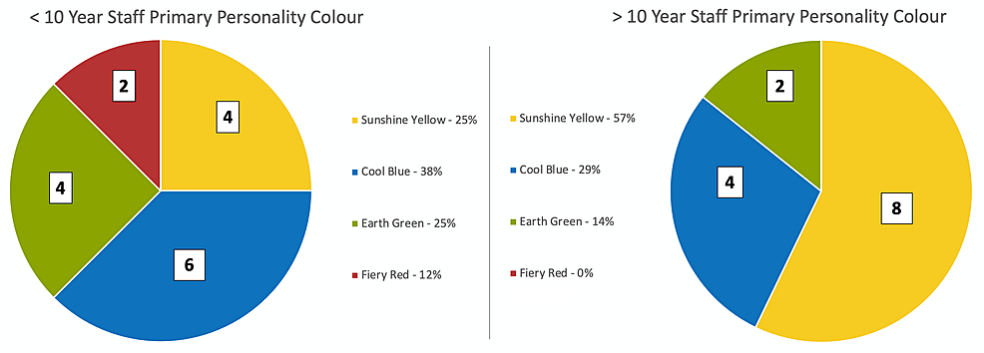
Proportion of physicians with less than and greater than 10 years of clinical experience with each personality color according to the Insights Discovery questionnaire.

Among the physician participants with less than 10 years of clinical experience, four of 16 participants (25%) were sunshine yellow, six (38%) were cool blue, four (25%) were earth green, and two (12%) were fiery red. Among the physician participants with more than 10 years of clinical experience, eight of 14 participants (57%) were sunshine yellow, four (29%) were cool blue, two (14%) were earth green, and zero (0%) was fiery red. As shown in Figure 4, less than compared to greater than 10 years of clinical experience showed no statistical significance between groups for each respective personality color.

## Discussion

Using the Insights Discovery tool, we aimed to understand the landscape of personality profiles in a pilot population of emergency physicians. There was a disproportionately low amount of fiery red personality types among the study population (7%, p=0.02), indicating a possible paucity of emergency physicians possessing these personality characteristics. Also of note, none of the greater than 10-year staff subgroup had a fiery red personality type. While this was a small subgroup sample, it may suggest a trend towards shifting in personality type with years of experience. Otherwise, a diverse landscape of personality types existed, including introversion, extroversion, relationship-oriented, and task-oriented preferences.

While this was the first known publication of personality characteristics using the Insights Discovery assessment tool, previous studies have indicated a mosaic of personality characteristics among emergency physicians. Boyd and Brown found that using the Myers-Briggs tool, the most common personality group in their cohort was the Extrovert/Intuitive/Thinking/Judging (ENTJ) type, though no statistical significance was assessed [4]. Pajonk et al. used the Hamburg Personality Inventory, finding that no homogenous characteristics exist between emergency physicians, or between emergency physicians and paramedic colleagues [5].

This pilot study used an innovative, easy-to-administer, and validated personality assessment tool to demonstrate the feasibility of its use in a study format. Furthermore, participants were able to debrief their personality assessment with Insights Discovery, allowing for understanding and interpretation of their results for a translational benefit to their future practice. Despite these strengths, it is difficult to draw meaningful large-scale conclusions from a sample of 30 participants by convenience sampling. Furthermore, the use of multiple different personality assessment tools in the literature makes a meaningful comparison between studies difficult. Finally, selection bias may be present, as certain personality types may be more likely to volunteer for study participation over others.

Self-awareness activities are an important tool for physicians to improve their communication strategies and team functioning within the emergency department. This study provides feasibility for future large-scale personality assessment initiatives. Further expansion to involve allied health professionals might improve our ability to deliver care via enhanced understanding and awareness of our working teams.

## Conclusions

Emergency departments include a large and diverse group of physicians dedicated to a common goal, providing high-quality patient care. By administering Insights Discovery, a better understanding of the team on both individual and departmental levels can be gained. Physicians receive valuable self-assessment to incorporate into their practice, while the collection of department-wide epidemiologic data can inform department educators and administrators about the unique makeup of the group. Overall, this study is the first of its kind in North America, shedding light on the diverse personality characteristics of physicians within the unique emergency department environment.
